# Editorial: Methods and applications in invertebrate physiology

**DOI:** 10.3389/fphys.2023.1158972

**Published:** 2023-02-17

**Authors:** Natraj Krishnan, Tetsuya Tanaka, Graziano Fiorito, Fernando Ariel Genta, Pamela Imperadore

**Affiliations:** ^1^ Department of Biochemistry, Molecular Biology, Entomology and Plant Pathology, Mississippi State University, Starkville, MS, United States; ^2^ Laboratory of Infectious Diseases, Joint Faculty of Veterinary Medicine, Kagoshima University, Korimoto, Kagoshima, Japan; ^3^ Department of Biology and Evolution of Marine Organisms, Stazione Zoologica Anton Dohrn, Naples, Italy; ^4^ Laboratório de Bioquímica e Fisiologia de Insetos, Instituto Oswaldo Cruz, Fundação Oswaldo Cruz (IOC/FIOCRUZ), Rio de Janeiro, RJ, Brazil; ^5^ Department of Biology and Evolution of Marine Organisms, Stazione Zoologica Anton Dohrn, Napoli, Italy

**Keywords:** invertebrate animal models, invertebrate physiology, *Drosophila melanogaster*, RNAi—RNA interference, cephalopod, gene expression, fluorescent *in situ* hybridization (FISH), *Bombyx mori*

Interest and research on invertebrate animal models has increased over the past decades and new methods and applications are being developed leading to fundamental physiological discoveries with applications ranging from biology to medicine. Invertebrate study systems thus have become cornerstones of biological and biomedical research, providing key insights into fields from genetics to behavioral ecology. Species being investigated and used as models range from terrestrial invertebrates such as insects and nematodes to freshwater and marine life including crustaceans, molluscs and many others. While the use of *Drosophila* for genetic studies was established in the early 20th century, it has since been used for many other applications including developmental biology, stem cell biology, endocrine function and metabolism, innate immunity, neurobiology, toxicology, etc. (Beckingham et al., 2005; Gilbert, 2008). Similarly, the nematode *Caenorhabditis elegans* has been utilized as a model system to investigate biological and physiological processes common to all animals (Strange, 2007). In recent years, there has been an increased use of other invertebrates to both understand the evolution of these organisms but also to throw light on developmental processes in higher animals (Holland and Gibson-Brown, 2003; Bicker, 2005; Darling et al., 2005; Swalla, 2006; Bicker, 2007; Arendt et al., 2008; Wessel et al., 2010; Wilson-Sanders, 2011; Castillo and de la Guardia, 2017).

This Research Topic focuses on some of the recent advances in Methods and Applications in Invertebrate Physiology and is a compilation of ten original research articles dealing with methods (six) or research (four) and one review article (a total of eleven articles) dealing with invertebrate physiology.

Sellamuthu et al., describe a method for selecting reference genes for normalizing gene expression results for different experimental conditions for an important wood-boring destructive coleopteran beetle *Ips sexdentatus*. Currently practiced management strategies have proven inadequate to stem the outbreak of bark beetle populations, which necessitates investigations into novel mitigation strategies using state-of-the-art molecular methods. In this context, gene expression and functional genomics studies are crucial to generate alternative strategies. This work represents the first reference gene validation study in *I. sexdentatus*, an ecologically important wood-boring beetle which provides valuable information on reference genes for future molecular studies on host-beetle interactions and functional genomic studies on this bark beetle or other Ips beetles (Coleoptera: Curculionidae: Scolytinae).

In another study on identification and validation of reference genes for beetles of the genus *Monochamus* that serve as vector for the pine wood nematode (PWN) *Bursaphelenchus xylophilus*, Li et al., evaluate the stability of fourteen candidate reference genes in *Montipora saltuarius*. This was conducted at different developmental stages associated with infection of PWN or PWN treatment conditions and was evaluated using delta Ct, geNorm, NormFinder, BestKeeper and RefFinder algorithms. Based on this study, *RPL7* and *RPS5* were considered the most stable reference genes in the pupae treated with PWN. *RPS5* and *SNX6* could be used as reference genes in the adults treated with PWN. *RPL7*, *EF1-γ*, and *RPS5* could be used as stable reference genes in all the samples. This study lays the ground-work for understanding the phoretic relationship between *M. saltuarius* and *B. xylophilus*.

Restoration of reef building corals are crucial for a healthy marine ecosystem. Guo et al., provide a possible solution for the decline in reef building corals due to climate change, ocean acidification and also by invasive species. They report on the sequencing of the transcriptome of the fibroblast growth factor (FGF) and its receptor (FGFR) involved in coral budding morphogenesis of four common and dominant reef building corals *Pocillopora damicornis*, *Montipora capricornis*, *Acropora muricata*, and *Pocillopora verrucosa*. Phylogenetic analysis revealed that FGF8 and FGFR3 are widely distributed in hydrozoan animals. Employing three dimensional models of FGF8 and FGFR3 as well as reconstruction of binding models, they found that FGFR3 is a tyrosine kinase receptor and the ligand is FGF8 in *A. muricata*, *P. damicornis*, and *Montipora capricomis*, but not in *P. verrucosa*. In *P. verrucosa* FGF8 is not the ligand for FGFR3 but its receptor needs activation by other TK-ligands. Given this understanding of the morphogenesis signaling mechanism, they postulate the possibility of using biological agents that could activate morphogenesis as a restorative measure for reef building corals.

Zabelina et al., provide a method that determines the exact timing of microinjection of silkworm *Bombyx mori* L embryos to generate successful pathenogenetic clonal lines. This technique addresses a critical hurdle in utilizing parthenogenetic strains of silkworms in transgenesis and increases efficiency of transgene injection and survival. The authors determine 18 h after egg activation as the optimal time for transgene microinjection and also provide a detailed troubleshooting guide to help other researchers improve the survival rate of injected embryos.

RNA interference (RNAi) strategy is a strategy that is being widely used by researchers to knock-down specific genes to interrogate their function in life science research as well as has potential to be used in pest management strategies. A major obstacle to this technique is the generation of large quantities of double stranded RNA (dsRNA) in an economic and cost effective manner. Verdonckt and Vanden Broeck compare and report on methods for large-scale production of high-quality dsRNA from the *E. coli* HT115 bacterial system. They also determine the efficiency of the dsRNAs thus produced at inducing knockdowns in a lepidopteran cell line. It is expected that these results will provide researchers with an efficient and cost effective method to generate dsRNA for their knock-down studies.

The major component of insect cuticle is the polysaccharide chitin. To understand cuticle metabolism and biology, it is important to study chitin structure in chitin deficient phenotypes of insects. Current methods rely on expensive and labor intensive electron microscopy studies. To circumvent this, Flaven-Pouchon and Moussian describe a fluorescent-microscopy based technique that leverages utilizing the common polysaccharide marker Fluorescent brightener 28 (FB28) in whole-mount *Drosophila melanogaster*. To ensure effective penetration, they recommend performing the staining at 65°C. This technique will have applicability to a large variety of insects.

*In situ* hybridization enables the detection and precise localization of a specific nucleic acid sequence within an individual cell. *In situ* hybridization (ISH) combines three main advantages: great sensitivity, precise anatomical localization, and the possibility of quantification. It is extensively used in evolutionary and developmental biology. Paganos et al., describe a rapid, reliable and accurate fluorescent *in situ* hybridization (FISH) protocol that can be utilized for a variety of marine species—five species of echinoderms, species representatives of the mollusks, tunicates and cephalochordates. This protocol is expected to be of great utility to the Evo-Devo community with the possibility of being extended to other non-marine species.

Elagoz et al., provide an optimized protocol for visualizing and imaging Octopus embryo neurogenesis. A major challenge to visualizing and investigating organ functioning is the ability to effectively detect gene expression patterns in whole organ or embryo. Here, the authors describe a whole mount multiplexed RNA *in situ* hybridization chain reaction version 3.0 (HCR v3.0) in combination with immunohistochemistry (IHC), followed by fructose-glycerol clearing and light sheet fluorescence microscopy (LSFM) imaging on *Octopus vulgaris* embryos. The authors hypothesize that the developed experimental pipeline can be adapted to other model and non-model organisms.

The Cephalopod digestive tract physiology has been the Research Topic of several studies particularly because of its importance from the standpoint of contributing to comparative and evolutionary studies. In their comprehensive review on the physiology of the digestive system of *O. vulgaris*, Andrews et al., describe a variety of techniques, gaps that exist in knowledge and future research areas in this aspect.

With the inclusion of cephalopods in the legislation related to ethical use of animals for experimental purposes, handling cephalopod mollusks in research is challenging and sedation and/or anesthesia is necessary whenever more invasive procedures are required. Sprecher et al., test the physiological effects of ethanol and magnesium chloride as anesthetics in the squid *Loligo vulgaris* embryos. The authors conclude that ethanol had a faster onset of action and faster recovery than magnesium chloride, being potentially more adequate as an anesthetic for shorter procedures. Also, the authors suggest that the late developmental stages of *L. vulgaris* embryos could represent a good model to evaluate anesthetics for cephalopods.

The neural control of coordinated limb movements have long been a Research Topic of investigations in invertebrates as well as vertebrates. Insects have served as preeminent models in such studies. Hammel et al., investigated the mechanisms underlying curve walking in the stick insect *Carausius morosus* during optomotor-induced turning. This study presents evidence that the motor output to the three main leg joints of the meso- and the metathoracic legs of the stick insect, during turning is not only joint-specific, but also differs depending on the thoracic segment. Also, changes in the activity during turning are most likely mediated by influences on local central pattern generating network (CPG) activities, and the respective influences on segmental CPGs weaken caudally towards the metathorax. The authors conclude that the turning-related motor output strongly depends on local or inter-leg sensory feedback.

In conclusion, the articles in this Research Topic e-collection is a compilation of contributions of different experts in the field of invertebrate physiology and their applications, in a wide variety of invertebrate species. These articles address specific methods as well as extend our knowledge while providing new insights and perspectives in the field of invertebrate physiology.
